# Addressing Heterogeneity in Direct Analysis of Extracellular Vesicles and Their Analogs by Membrane Sensing Peptides as Pan‐Vesicular Affinity Probes

**DOI:** 10.1002/advs.202400533

**Published:** 2024-05-31

**Authors:** Alessandro Gori, Roberto Frigerio, Paola Gagni, Jacopo Burrello, Stefano Panella, Andrea Raimondi, Greta Bergamaschi, Giulia Lodigiani, Miriam Romano, Andrea Zendrini, Annalisa Radeghieri, Lucio Barile, Marina Cretich

**Affiliations:** ^1^ Consiglio Nazionale delle Ricerche Istituto di Scienze e Tecnologie Chimiche “Giulio Natta” (SCITEC) Milano 20131 Italy; ^2^ Cardiovascular Theranostics Istituto Cardiocentro Ticino Ente Ospedaliero Cantonale Via Tesserete 48 Bellinzona CH‐6500 Switzerland; ^3^ Institute for Research in Biomedicine Faculty of Biomedical Sciences Università della Svizzera italiana (USI) Bellinzona CH‐6500 Switzerland; ^4^ Department of Molecular and Translational Medicine University of Brescia Viale Europa 11 Brescia 25123 Italy; ^5^ Center for Colloid and Surface Science CSGI Florence 50019 Italy; ^6^ Euler Institute Faculty of Biomedical Sciences Università della Svizzera Italiana Lugano 6900 Switzerland

**Keywords:** cardiovascular disease, digital detection, exosomes, extracellular vesicles, isolation, peptides, single molecule array

## Abstract

Extracellular vesicles (EVs), crucial mediators of cell‐to‐cell communication, hold significant diagnostic potential due to their ability to concentrate protein biomarkers in bodily fluids. However, challenges in isolating EVs from biological specimens hinder their widespread use. The preferred strategy involves direct analysis, integrating isolation and analysis solutions, with immunoaffinity methods currently dominating. Yet, the heterogeneous nature of EVs poses challenges, as proposed markers may not be as universally present as thought, raising concerns about biomarker screening reliability. This issue extends to EV‐mimics, where conventional methods may lack applicability. Addressing these challenges, the study reports on Membrane Sensing Peptides (MSP) as pan‐vesicular affinity ligands for both EVs and their non‐canonical analogs, streamlining capture and phenotyping through Single Molecule Array (SiMoA). MSP ligands enable direct analysis of circulating EVs, eliminating the need for prior isolation. Demonstrating clinical translation, MSP technology detects an EV‐associated epitope signature in serum and plasma, distinguishing myocardial infarction from stable angina. Additionally, MSP allow analysis of tetraspanin‐lacking Red Blood Cell‐derived EVs, overcoming limitations associated with antibody‐based methods. Overall, the work underlines the value of MSP as complementary tools to antibodies, advancing EV analysis for clinical diagnostics and beyond, and marking the first‐ever peptide‐based application in SiMoA technology.

## Graphical abstract

Membrane Sensing Peptides (MSP) serve as pan‐vesicular affinity ligands in a “one bead – multiple markers” approach, enabling direct EV analysis in serum and plasma. MSP technology is showcased in distinguishing myocardial infarction from stable angina and allows analysis of tetraspanin‐lacking vesicles such as Red Blood Cell‐derived EVs. MSP enhances EV analysis, marking the first peptide‐based application in SiMoA technology.

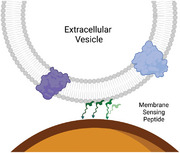

## 1 Introduction

Extracellular vesicles (EVs) are nanosized, membrane‐bound particles released by cells into the extracellular space^[^ ^1^ ^]^ and are known to play essential roles in cell‐to‐cell communication.^[^ ^2^, ^3^ ^]^ EVs are arising unparalleled expectations in the diagnostic field, given their capacity to enrich potential protein biomarkers which otherwise constitute only a very small portion of the total proteome of body fluids (<0.01%).^[^ ^4^, ^5^, ^6^, ^7^, ^8^ ^]^ The putative diagnostic power of EVs is particularly compelling for those biological specimens rich in EVs, including blood and urine, which allow for a non‐invasive sample collection with respect to tissue biopsies. However, despite considerable efforts, EVs isolation from complex body fluids remains arduous, time‐consuming, and difficult to standardize. Combined with a growing body of evidence that pre‐analytic steps in EVs sample preparation can influence^[^ ^9^, ^10^ ^]^ the downstream analysis, multistep protocols for EVs analysis remain unsuitable for large biobanks screening and even less applicable in routinary diagnostic settings. As such, arguably, only integrated isolation‐and‐analysis workflows could enable the translation from research settings to the real usage of EV‐associated biomarkers into clinical viable practices, with microfluidics^[^ ^11^, ^12^, ^13^, ^14^ ^]^ and bead‐based systems^[^ ^15^, ^16^, ^17^ ^]^ appearing as the most promising methods.

In these systems, immunoaffinity remains the standard to enrich EVs prior to analysis, with typically used targets for isolation including tetraspanins (CD9, CD81, CD63) and other EV‐surface proteins such as EpCAM or EGFR. However, with the surge of sorting methods and single‐vesicle analysis techniques,^[^ ^18^ ^]^ the high heterogeneity of EVs is clearly emerging: several markers proposed to be ubiquitous are less prevalent than believed, and multiple biomarkers patterns concur in single vesicles but only in small sub‐fractions.^[^ ^19^, ^20^, ^21^ ^]^ Thus, affinity‐based enrichment of EV subpopulations based on specific surface proteins lacks of a comprehensive view on circulating EVs and, if not coupled to other techniques (e.g., proteomics), can lead to missing or misleading information, undermining the reliability of downstream biomarker discovery programs. It is paradigmatic that a recent work reported on the loss of up to the 80% of EVs with diagnostic potential when affinity isolation by single tetraspanin protein is used, and a loss of 36–47% when a tetraspanin cocktail is employed.^[^ ^22^ ^]^

This issue reverberate also on the exponentially growing field of engineered,^[^ ^23^ ^]^ synthetic^[^ ^24^ ^]^ or hybrid EV‐mimetics and analogs^[^ ^25^, ^26^ ^]^ and other bacterial^[^ ^27^, ^28^ ^]^ or plant‐derived bio‐nanoparticles,^[^ ^29^ ^]^ which are under extensive investigation as the next generation nano delivery systems in the therapeutic arena. Indeed, their successful translation depends, among other factors, also on the availability of high‐sensitivity characterization methods to be applied to robust QC protocols, and to assess biodistribution parameters including their concentration and clearance in biofluids. In this frame, current antibody‐based methods developed to analyze natural EVs may lack applicability due to limited to no knowledge of “unnatural” EV markers.

**Figure 1:**
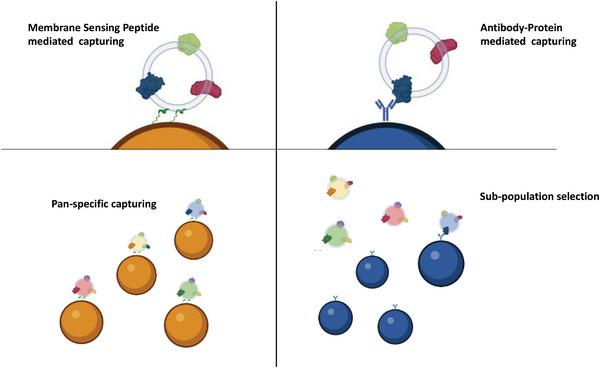
Membrane Sensing Peptides (MSP) – left panel – are able to capture small EVs or analogs based on their membrane physical traits, irrespectively from surface protein abundance, rendering a pan‐specific capturing. On the contrary, antibody‐mediated capturing – right panel – depends on the presence of specific protein epitopes on the EV surface, selecting only specific sub‐populations.

Building on these rationales, we have previously conceptually introduced and provided preliminary proof‐of‐concept^[^ ^30^, ^31^, ^32^ ^]^ in the use of Membrane Sensing Peptides (MSP) as a class of affinity ligands working as pan‐vesicular probes for nanosized lipid particles, including small EVs (sEVs, <200 nm size range), irrespective of their natural or synthetic origin. Unlike antibodies, MSP shows specific affinity for the highly curved lipid membrane, which can be considered a shared “epitope” for nanovesicles, making MSP probes working agnostically in regard to the nature and relative abundance of surface proteins (**Figure** **1**). In general, curvature sensing is a well‐known process resulting from the presence of lipid packing defects characteristic of highly tensioned membranes, which favors the insertion and binding stabilization of amphipathic protein domains (or peptides) within the lipid bilayer.^[^ ^33^, ^34^, ^35^, ^36^ ^]^ Overall, the use of MSP probes is conceptually complementary to that of antibodies, opening new perspectives in addressing some of the current limitations plaguing EV analysis.

Here, we expand our previous findings and we provide full evidence in the use of a bradykinin‐derived MSP ligand (Bk‐MSP, RPPGFSPFR‐RPPGFSPFR)^[^ ^30^ ^]^ for the integrated isolation‐and‐analysis (direct analysis) of circulating EVs in blood derivatives (serum and plasma) in a clinically compliant workflow, without the need for prior EV isolation. Besides functional efficiency from previous works, Bk‐MSP was selected due to its high solubility and no propensity for aggregation, which were foreseen as a possible limit for other amphipathic MSP peptides in microscale applications. Specifically, we relied on a streamlined process that integrates on‐bead capture and vesicle phenotyping through the Single Molecule Array (SiMoA)^[^ ^37^, ^38^ ^]^ technology. The ultrasensitivity of this digital detection platform, integrated into the high‐throughput Simoa instruments, favored its diffusion in clinical centers for monitoring of biomarkers in the field of neurological diseases, cancer and other chronic diseases. Recently, SiMoA significant clinical potential have been largely documented in EV analysis,^[^ ^39^, ^40^, ^41^, ^42^ ^]^ making it arguably one of the most promising platforms that meets the criteria for facilitating the clinical translation of EVs.

Overall, the efficiency of MSP probes is shown in sampling EVs directly from serum and plasma, with minimal carry over of contaminants and allowing subsequent EV epitope analysis in a one‐step process. We additionally demonstrate possible translation in clinical settings by directly analyzing an EV‐associated epitope signature in a cohort of serum and plasma samples for stratification of patients with myocardial infarction versus stable angina. Of note, MSP‐based assays enabled the assessment of the value of an EV‐associated putative marker that was not enriched in the tetraspanin‐responsive EV subpopulation. Moreover, to further demonstrate the versatile and unique capabilities of MSP probes, we extend this approach to Red Blood Cell derived EVs (RBC‐EVs),^[^ ^43^ ^]^ that we used as a model for EV analogs lacking the canonical protein markers profile.^[^ ^44^ ^]^ Last, this work reports on the first ever peptide‐based assay in the SiMoA technology.

## 2 Results and Discussion

### 2.1 MSP Selectivity of EV Binding in Serum and Plasma

Blood is a highly complex matrix that proved to be challenging for reproducible EV isolation and biomarker analysis.^[^ ^9^ ^]^ The combination of more than one purification method, for example density cushion and size exclusion chromatography, is often needed to efficiently enrich EVs from other blood components, mostly including lipoproteins, which largely outnumber EVs.^[^ ^45^, ^46^, ^47^ ^]^ Yet, gold standard techniques for EV pre‐isolation, or combination of orthogonal purifications, are impracticable for large cohort studies or routine diagnostics.

As such, the integration of EV isolation and analysis in a one‐step streamlined protocol is a highly appealing strategy to pave the way to actual clinically compliant procedures. In view of its direct use in blood specimen without pre‐isolation steps by the SiMoA Bead Technology, that we have previously explored for digital EV immune‐phenotyping,^[^ ^48^ ^]^ we set to preliminary assess whether Bk‐MSP meets the criteria of specificity of EV capture with respect to common contaminants in blood‐derived specimen.

**Figure 2:**
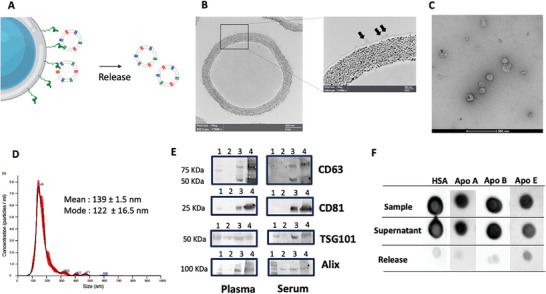
A) scheme of the catch and release strategy here applied to demonstrate efficient isolation of small EVs, their release in intact conditions and negligible presence of contaminants after separation. B) TEM image of agarose beads and surface captured EVs (Black arrows). C) TEM image of EVs captured from serum after release with 0.5 m imidazole, showing intact, membrane‐enclosed vesicles. D) Representative NTA analysis (three technical replicates) of EVs captured from serum and released after imidazole treatment. The red line represents the standard deviation of the medium from three analysis. Overestimation of EV diameter by NTA could be ascribed to instrument inability to detect EVs <70 nm. E) Western Blot detecting typical EV‐associated surface (CD63 and CD81) and luminal (TSG101 and Alix) markers in the released particles (Lane 3) after capturing from Plasma (left panels) and Serum (right panels). Lane 1: Molecular Marker. Lane 2: Red Blood Cell EVs (negative control for tetraspanin) Lane 4: Commercial standard of HEK cells derived EVs (positive control). Thirty‐two microliters of sample were loaded in each well. F) Immune dot‐blot analysis to check presence of common contaminants: Human Serum Albumin (HSA) Apolipoproteins A, B, and E (Apo A, Apo B, Apo E respectively) in the starting Sample (Serum), in the Supernatant after capturing and in the Released EV fraction. Negligible contaminant signals are detectable after EV isolation. Results from other pre‐analytical conditions tested, plasma‐EDTA, plasma‐heparin and plasma‐citrate are shown in Figure S1 (Supporting Information).

In this frame, while it was out of the scopes of the present work to deliver an optimized tool for quantitative EV isolation from blood samples, we applied a Bk‐MSP‐based protocol that we recently proposed for EV isolation from cell‐conditioned medium by capture and release from modified agarose beads (**Figure** **2**A, see Experimental Section), and the resulting preparations were analyzed according to MISEV guidelines.^[^ ^1^ ^]^ This served to introduce an early level of control before moving to integrate MSP on the SiMoA platform, and later enabled us to benchmark and confidently match results obtained by direct ultrasensitive analysis with traditional characterization techniques.

After capturing on Agarose beads (Figure 2B), the integrity, size, and morphology of released EVs were verified by TEM (Figure 2C) and Nanoparticle Tracking Analysis (Figure 2D), whereas Western Blot (Figure 2E) was used to detect the presence of typical EV‐associated surface tetraspanins (CD63 and CD81) and luminal (TSG101 and Alix) markers. Red Blood Cell derived EVs were used as negative control for tetraspanins, while a commercial standard of HEK cell‐derived EVs was used as positive control for all markers. Released EVs analyzed by TEM images showed membrane‐enclosed vesicles compatible with the size distribution measured by NTA (Figure 2D). NTA reported a mean size of 139 ± 1.5 nm, representing the average value of EVs recovered after agarose beads isolation, whereas the most frequently occurring EV size was shown by a calculated mode of 122 ± 16.5 nm. It is well known that, with respect to TEM analysis, NTA tends to overestimate EV distribution size. Detection of characteristic molecular markers (CD63, CD81, TSG101, and Alix) by Western Blot (Figure 2E) confirmed the EV nature of released particles (Lane 3) in adherence with the minimal information requirements on EV research recommended by the International Society for Extracellular Vesicles (ISEV).^[^ ^1^ ^]^ Most importantly, the binding selectivity versus the most abundant blood “contaminants” in EVs analysis, including lipoproteins (Apolipoprotein A, Apolipoprotein B, Apolipoprotein E) and albumin, was assessed by immune‐dot blot analysis. Different pre‐analytical conditions in blood collection were tested as they are well known to strongly influence downstream results.^[^ ^49^ ^]^ Findings reported in Figure 2F, show negligible signals for contaminants in the serum EVs released fraction. Similar results were obtained with plasma, independently from the pre‐analytical conditions (Figure S1, Supporting Information) confirming applicability of MSP EV capturing in all the pre‐analytical conditions tested (plasma‐EDTA, plasma‐heparin, and plasma‐citrate) and for both plasma and serum based analytical workflows.

Overall, the MSP‐based isolation protocol was used to provide preliminary evidence that MSP probes were eligible candidates for direct EV analysis in blood derived specimen, with minimal matrix and pre‐analytical conditions interference.

**Figure 3:**
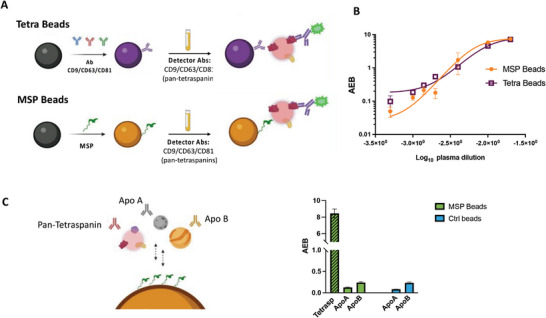
A) Scheme of SiMoA beads functionalization by a combination of antibodies directed against CD9/CD63/CD81 tetraspanin (Tetra Beads) and by MSP (MSP beads). Both bead types were used in a pan‐tetraspanin detection of EVs from plasma samples. B) Average Enzyme per Bead (AEB) signal obtained using Tetra beads and MSP beads in a SiMoA three‐step assay of serial dilutions (from 1:50 to 1:2000) of a pool of plasma from healthy donors. C) Assay designed to assess possible non‐specific interaction of lipoproteins onto SiMoA MSP beads (Left panel). A three‐step assay for plasma (dilution 1:100) EV analysis was performed using either CD9/CD63/CD81, anti‐Apolipoprotein A, or Apolipoprotein A as detector antibodies. The obtained AEB signals (right panel) show negligible interaction of MSP beads with lipoproteins in comparison with the signal obtained for tetraspanin (logarithmic scale). Notably, signals for lipoprotein interaction are similar to those obtained with control beads (non‐functionalized).

As such, we then moved to set up an integrated assay for EV direct analysis in human plasma on the SiMoA platform. MSP probes were conjugated to SiMoA microbeads through a customized protocol (see Experimental Section and Supporting Information) and used in a direct immunoassay for EVs using a combination of detector antibodies targeting CD9, CD63 and CD81 (pan‐tetraspanin detection) as shown in **Figure** **3**A. As a reference, an analogous assay was set up by modifying SiMoA beads with a combination of anti CD9, CD63, and CD81 antibodies (pan‐tetraspanin beads, hereafter referred to as Tetra beads) (Figure 3A). These served as a proxy of global capture beads for tetraspanins‐positive EVs. MSP beads and Tetra beads were subsequently used for EV pan‐tetraspanin detection directly in serial dilutions of a pool of plasma samples from healthy controls using a SiMoA three step assay (Experimental Section). Average Enzyme per Bead (AEB) signals at each plasma concentration, for both types of beads, are reported in Figure 3B. Overall the two systems performed comparably, enabling the detection of tetraspanin‐positive EVs directly from plasma (without pre‐isolation steps) within a dilution range from 1:50 to 1:2000. Notably, MSP beads provided a wider dynamic range of usage.

To further investigate the specificity of MSP probes for EV capturing from plasma, we assayed possible non‐specific interactions of lipoproteins onto SiMoA beads (Figure 3C). To this aim, we set an assay for immune‐detection of lipoproteins non‐specifically bound onto MSP beads using anti Apolipoprotein A (ApoA) and B (ApoB) as detector antibodies after incubation of plasma diluted 1:100 (see Experimental Section). Results are reported in Figure 3C (right panel) showing AEB signals for both ApoA and ApoB that are negligible in comparison with corresponding pan‐tetraspanin signal derived from EV capturing from the same plasma sample (logarithmic scale for Y axis). Notably, control beads (without MSP functionalization) provided a similar level of non‐specific interaction with lipoproteins.

It is worth underlining that the use of MSP differs from other pan‐EV binding approaches, including charged‐based systems^[^ ^50^ ^]^ or purely hydrophobic probes,^[^ ^51^ ^]^ which lack selectivity. MSP affinity involves a two‐step process: an initial electrostatic‐driven interaction with the membrane followed by insertion, folding and binding stabilization (anchoring) mediated by peptide hydrophobic residues. We speculate that this unique mechanism adds an extra level of selectivity compared to mere charge‐ or lipid‐based interactions. In addition, while not in current work scopes and not here further explored, this opens interesting perspectives in Bk‐MSP tools for EVs isolation from blood samples propaedeutic to other analysis or use.

### 2.2 MSP Capture Returns Representative EV Markers Abundance Profile

Prior to application into real context scenarios, we more deeply investigated the MSP representativeness in EV binding propaedeutic to immunophenotyping, taking as a reference EVs with “canonical” surface marker profile. In other words, by testing three markers of consolidated use (CD9/CD63/CD81), we aimed to assess whether the outcome of analysis obtained by using MSP probes is reliable and results in no specific enrichment of some EVs subpopulations. Specifically, we set to verify that the EVs capture mediated by MSP returns the same level of single tetraspanins (CD9/CD81/CD63) relative abundance with respect to that provided by the corresponding antibodies. This was performed straightway into plasma from six healthy donors and in compliance with the direct analysis protocol reported above. As a reference, SiMoA microbeads were functionalized with a combination of antibodies directed against CD9, CD63, CD81 tetraspanins (Tetra beads). These would serve as a proxy of global and unbiased capture beads for tetraspanins‐positive EVs, regardless of the relative abundance among the three markers.

**Figure 4:**
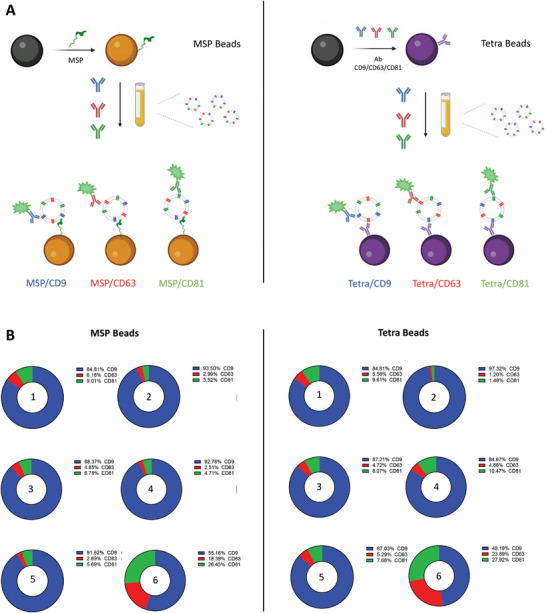
A) SiMoA Beads were modified by MSP (left panel) and by a combination of antibodies directed against CD9/CD63/CD81tetraspanin (right panel). Single tetraspanin immune‐phenotyping of plasma EVs from 6 healthy donors was run in parallel on the two types of beads. B) For both settings and each sample, the total AEB (Average Enzyme per Bead) is calculated as the sum of single CD9/CD63/CD81 AEB detection. Single tetraspanin expression level is calculated as the AEB% over the total AEB. Both methods confirm the expected heterogeneity of EV samples with remarkable accordance of the two systems in terms of differential tetraspanin profiling. AEB data and %CV are reported in Table S1 (Supporting Information).

We then compared results obtained by alternatively using MSP beads versus Tetra beads (**Figure** **4**A) for EVs capture, followed by surface immune‐phenotyping to detect CD9/CD63/CD81 tetraspanin individually.

Overall, an almost overlapping pattern of each tetraspanin relative abundance was observed either by using MSP beads (Figure 4B, left panel) or Tetra beads (Figure 4B, right panel).

The analyzed context suggests that MSP binding does not result in a biased selection of EVs, nor that specific subtypes are enriched, and thus confirms their representativeness and reliability toward their perspective use in blind EV‐associated biomarker discovery programs.

In these regards, it is worth noticing that, even if in this specific context (plasma EVs) a pan‐tetraspanin capture is apparently well performing, it could not be the case for other EV samples where tetraspanins are poorly expressed and/or alternative abundant surface markers are not known.

Moreover, the relative abundance of CD9, CD63, CD81 highlighted the (expected) sample heterogeneity and the well‐known uneven distribution of the three proteins^[^ ^19^ ^]^ (Figure 4B). To the best of our knowledge, the vast majority of published work in EV analysis after immune‐based enrichment makes use of individual anti‐tetraspanin probes, without a prior pre‐assessment of their relative abundance. Also, this approach inherently returns the expression levels of those EV‐markers under investigation only in the specific, pre‐selected subpopulation, rather than providing information on their abundance frequency across different EVs. This poses concerns about the possible biases that can be introduced in downstream processes, thus reinforcing the need for alternative and complementary enrichment methods not based on EV subtypes pre‐selection.

To further illustrate this concept, we performed immune dot blot analysis of additional EV markers after EV depletion via immunocapturing experiments in plasma samples (Figure S2A, Supporting Information). We made use of SiMoA paramagnetic beads functionalized with MSP, as well as individual CD63, CD9, and CD81. Three extra markers (Mitofillin, CD41, MHC II) were selected based on previous studies that highlighted their varying abundance in tetraspanin‐responsive EV subpopulations.^[^ ^52^ ^]^ TSG101 was also included as control. It is noteworthy that all chosen markers exhibited only partial and/or tetraspanin‐dependent depletion when antibody beads were used, whereas MSP demonstrated evidence of consistent binding for each EV subpopulation (Figure S2B, Supporting Information). Interestingly, following MSP depletion, partial escaped capture could be detected only for EV subpopulations bearing Mitofillin and, to a very limited extent, CD41. This is consistent with the reported presence and enrichment of these markers in large EV subpopulations^[^ ^52^ ^]^ for which MSP would be expected to show less affinity.

Additionally, it is worth remarking that some circulating EVs do not generate from the endosomal biogenesis pathway (e.g., plasma membrane budding) and, as such, they lack or poorly express canonical tetraspanins (CD81, CD63, CD9). Red blood cells derived EVs (RBC‐EVs) were here used as a representative model for this class of EVs in MSP‐based analysis (see dedicated section below).

### 2.3 MSP in Tetraspanin‐Lacking EVs Samples

**Figure 5:**
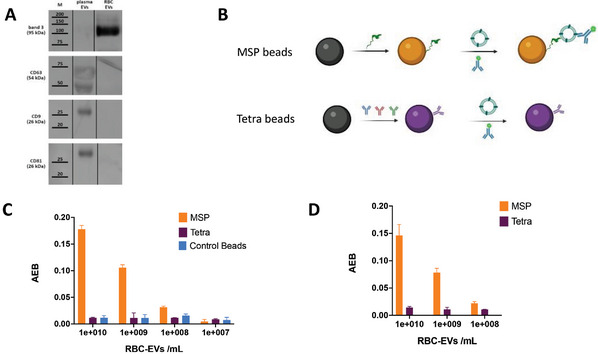
A) Western Blot of RBC‐EVs, in contrast to plasma isolated EVs, show poor expression of tetraspanin triad CD63, CD9, and CD81, reflecting their biogenesis pathway (budding from RBC plasma membrane). Legend: M = Marker; Plasma EVs: EVs isolated from plasma by MSP‐agarose modified beads; RBC‐EVs = RBC EVs ectosomes. B) SiMoA beads were modified by MSP and by a combination of CD9/CD63/CD81 antibodies (Tetra) and used to capture serial dilutions of RBC‐EV using anti‐Band 3 antibody for detection. C) Detection signals reported as Average Enzyme per Bead (AEB) subtracted of the corresponding blank signals showing the capacity of MSP beads to capture RBC‐EVs down to 10^8^ vesicles/mL with a clear dose‐response trend which is not detectable neither by Tetra beads, nor by control (unfunctionalized) beads D) Detection of RBC‐EVs when spiked in plasma.

To further illustrate one of the key advantages of the MSP technology, i.e., surface protein‐independent capturing, we selected EVs derived from Red Blood Cells (RBC‐EV) as a model for vesicles lacking canonical CD9/CD81/CD63 expression. The ultimate goal was to demonstrate that conventional (and commercially available) tools designed for tetraspanins will prove ineffective for their characterization, as well as for analysis purposes of many EV mimics and analogs. RBC‐EV well fit these purposes – they are indeed under investigation as candidates for drug delivery and other translational applications due to their high safety profile and minimal risk of horizontal gene transfer.^[^ ^44^ ^]^ RBCs lack the endolysosomal system hence they generate EVs only by plasma membrane budding (i.e., ectosomes), not expressing the canonical exosome tetraspanins (CD81, CD63, CD9) (**Figure** **5**A) but highly enriched in erythrocyte specific Band 3 anion transport protein (Band 3).^[^ ^53^, ^54^ ^]^ RBCs vesiculation is in vitro induced by calcium ionophore, which also triggers phosphatidylserine flipping from the inner to the outer membrane leaflet while boosting EV release.

MSP beads and Tetra beads were again compared in RBC‐EVs analysis on SiMoA (Figure 5B) using anti‐Band 3 as detection antibody and serial dilutions of an RBC‐EV sample. Results are reported in Figure 5C. MSP beads effectively captured RBC‐EVs, demonstrating a dose‐response signal. In contrast, Tetra beads and Control beads (not functionalized) exhibited neither a dose‐response correlation nor an appreciable signal‐to‐noise value. Noteworthy, MSP beads were able to capture RBC‐EVs when spiked into a plasma sample (Figure 5D) mimicking the analysis of non‐endosomal vesicle populations (ectosomes) lacking the canonical exosome markers even in complex samples.

It is worth underlining that, while RBC‐EV are well‐characterized to date and have allowed us to select a characteristic protein marker for phenotyping purposes (Band 3), other emerging EV analogs may not be as well‐characterized, lacking known distinctive surface protein markers. This would further complicate the selection of suitable analytical tools and reinforces the need for “general” EV ligands that enable downstream biomarkers screening.

### 2.4 MSP Enable Enhanced EV Marker Assessment in Clinical Settings

It was previously demonstrated that EVs analysis can reveal the very early stages of cell stress that precede cardiomyocytes death and the release of troponin, the biomarker in clinical use for the diagnosis of the acute coronary syndrome (ACS). More specifically, it was shown that significantly increased levels of CD9/CD81/CD63‐responsive EVs and of co‐localized vesicular CD42a, and CD62P antigens—endothelial and platelet‐related antigens— were a distinctive signature in serum samples from patients experiencing ST‐elevation myocardial infarction (STEMI), an acute coronary event that precedes myocardial injury.^[^ ^55^ ^]^

In this frame, while replicating the full clinical validation was out of the scope of the current work, we still aimed at validating the use of MSP probes in this clinically relevant context, using an integrated isolation‐and‐analysis protocol that would match a viable workflow for direct EVs analysis from blood‐derived specimen. In the proposed workflow, a common molecular ligand (MSP) will serve a “one bead – multiple markers” analytical approach. Besides assessing the technical feasibility, it was crucial to determine whether MSP‐based EVs epitope profiling would return the same diagnostic value obtained by the use of antibodies as EV‐binding probes, such as the one that was used and validated in the previous study by Burrello et al.^[^ ^55^, ^56^ ^]^

**Figure 6:**
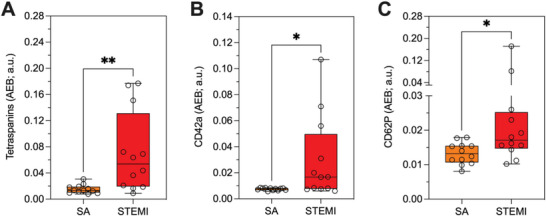
A) Expression of Tetraspanins CD9/CD81/CD63, B) CD42a and C) CD62P in serum of patients with ST‐segment elevation myocardial infarction (STEMI; red, n = 12), stable angina (SA; orange, n = 12). *t* Test: Tetraspanins – *p * = 0.002; CD42a – *p * = 0.018; CD62P – *p * = 0.014. Reported AEB were subtracted of the corresponding blank AEB signals: (Tetrasp: 0,0012; CD42a: 0,0011; CD62P: 0,0018). The study was conducted with MSP‐modified beads in a customized SiMoA assay as described in the Experimental Section. Analogous data for plasma samples are reported in Figure S4 (Supporting Information).

We therefore performed an MSP‐SiMoA assay for a selected panel of EV‐associated markers proposed to serve in STEMI diagnosis by probing both serum and plasma samples without any form of EVs pre‐isolation or enrichment. We evaluated the expression levels of CD9/CD81/CD63 tetraspanins (**Figure** **6**A) as well as those of co‐localized vesicular CD42a (Figure 6B) and CD62P (Figure 6C) antigens, and assessed their value in the stratification of patients experiencing STEMI (n = 12) versus those symptomatic with stable angina (SA, n = 12), who were not undergoing an acute ischemic event. These groups were carefully matched in terms of age, sex, and cardiovascular profile, utilizing a cohort previously described in the study by Burrello et al.^[^ ^55^ ^]^ Our findings revealed higher serum levels of all evaluated EV‐associated markers in serum of STEMI patients compared to SA (Figure 6).

To further validate the reliability of our approach in different starting materials (plasma versus serum), a correlation analysis in patients with STEMI or SA (Figure S4, Supporting Information) was performed for tetraspanins, CD62P, CD42a.

We observed a significant correlation for tetraspanins, and CD42a, but not for CD62P, in serum and plasma samples (with R values ranging between 0.575 and 0.677) (Figure S4, Supporting Information). Additionally, Bland Altman plots analysis demonstrated a consistent underestimation of the levels of expression of EV‐associated epitopes in serum compared to plasma (Figure S4, Supporting Information). No proportional or magnitude‐dependent biases were observed for tetraspanins and CD42a. However, CD62P appeared to be overestimated in serum compared to plasma for lower expression levels (I tertile = +134%) and underestimated for higher levels (II tertile = −188%; III tertile = −176%). This made it challenging to directly compare CD62P levels in the two biofluids. In this sense, we suggest that marker of activated platelets could be biased by pre‐analytical factors, due to uncontrolled platelet‐activation during plasma collection.

**Figure 7:**
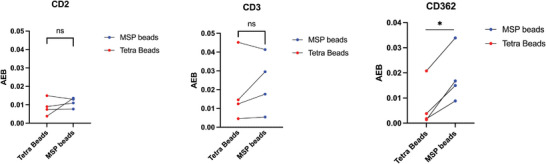
AEB signals for detection of CD2, CD3, CD362 in serum samples from STEMI patients (n = 4) using either MSP or Tetra beads in a SiMoA assay as described in the Experimental Section. While CD2 and CD3 AEB signals were not enhanced, CD362 signal resulted to be statistically different (higher) when using MSP beads. Reported AEB were subtracted of the corresponding blank signals: (CD2: 0,0015; CD3: 0,0017; CD362: 0,0011).

In our previous study,^[^ ^55^ ^]^ EVs analysis was inherently restricted to the epitope profiling of tetraspanin‐responsive EV populations. In this frame, the value of additional vesicular markers may be neglected or underestimated due to their absent or poor co‐localization with CD9/CD81/CD63, possibly reflecting also on analytical sensitivity. To prove that tetraspanin‐independent MSP binding of EVs may result in an additional level of information, we selected three markers that in the previous study did not show compelling diagnostic relevance. We then probed the presence of putative markers CD2, CD3, and CD326 on a small subset (n = 4) of STEMI samples in comparative assays using both Tetra beads and MSP beads (**Figure** **7**).

**Figure 8:**
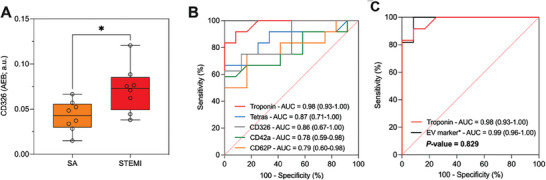
A) Expression of CD326 in serum of patients with ST‐segment elevation myocardial infarction (STEMI; red, n = 8), stable angina (SA; orange, n = 8). t Test CD326: *p * = 0.015.  The study was conducted with MSP‐modified beads in a customized SiMoA assay as described in the Experimental Section. B) ROC curve analysis for individual EV markers: CD9/CD81/CD63 tetraspanins (blue curve), CD42a (green curve), CD62P (orange curve), CD326 (grey curve), and hs Troponin (red curve). C) Diagnostic performance of combined EV markers (black curve) in comparison with the gold standard hs troponin (red curve).

Strikingly, even on this limited set of additional markers, the level of CD362 marker emerged as significantly higher in MSP‐based workflow with respect to Tetra beads (**Figure** **8**), as confirmed by paired t Test (p = 0,0071). This may suggest its poor co‐localization with CD9/CD81/CD63, and possibly account for its ineffective assessment in Burrello et al.^[^ ^56^ ^]^ This prompted us to expand the previous panel of vesicular markers (Figure 6) and screen CD362 in STEMI versus SA serum samples (n = 8) using MSP beads. Results shown in Figure 8 highlight a differential trend in CD362 expression between the two patient's cohorts at a statistically significant level, thus demonstrating that MSP‐based analysis can “restore” the informative value of markers that is otherwise overlooked in traditional tetraspanin‐based workflows.

The diagnostic performance of EV surface markers in discriminating STEMI patients and SA was assessed by ROC curve analyses (Figure 8B). In the training cohort, ROC curves indicated a high sensitivity for these markers. An aggregate marker including the four EV parameters (EV tetraspanin, CD62P, CD42a, and CD362 levels) was compared with classical high sensitive troponin assay (hs‐troponin) (Figure 8C). AUC confirmed excellent diagnostic performances of the aggregate marker (0.99; 95% CI: 0.96‐1.00), comparable with hs‐troponin alone (0.98; CI: 0.93‐1.000). Overall, we thus confirmed that using the proposed EV markers in a MSP‐SiMoA assay returned diagnostic performances not inferior to hs‐troponin (P‐value = 0.829).

## 3 Conclusion

Extracellular vesicles heterogeneity and their co‐existence with other components of the nanostructured secretome^[^ ^57^ ^]^ in complex biological matrices pose formidable challenges to their practical use in diagnostics. Likewise, the quest for suitable and broadly applicable analytical tools remains unfulfilled also for the emerging world of EV mimics and analogs. In general, novel tools and protocols for unbiased, streamlined, and clinically compliant (or QC) direct analysis of vesicular analytes are highly desirable and potentially game‐changing.

In this scenario, we have here reported on membrane sensing peptides (MSP) as an enabling tool for direct analysis of blood circulating EVs and their “non‐canonical” analogs. MSP represent an emerging class of affinity ligands able to enrich small vesicles on the basis of specific membrane biophysical traits, opposed to the pre‐selection of EV sub‐populations introduced by the use of antibodies.

As such, MSP present several advantages and complementarity to antibody‐based immunocapturing: i) MSP do not introduce biases in terms of sub‐population selection upstream of EV biomarker analysis; ii) MSP are suitable probes when there is limited knowledge of EV surface markers or no evidence of highly expressed proteins as proxies of general capturing; iii) MSP are not species‐specific and can be used for samples for which no validated antibodies exist (e.g., animals or bacteria); iv) MSP shares typical advantages of peptides over antibodies, such as cost‐effectiveness, longer shelf‐life, and no batch‐to‐batch variability.

In this study, MSP probes were initially validated for specificity and representativeness in capturing EVs from plasma and serum under diverse pre‐analytical conditions. This marks the first instance of a peptide‐based SiMoA assay integrated into a streamlined EVs direct analysis workflow. Subsequently, the use of MSP was validated in a clinically relevant context, building on previously published evidence emphasizing the significance of surface EV antigens in the context of cardiovascular diseases.^[^ ^56^ ^]^ Remarkably, MSP‐based analysis returned a diagnostic performance of EV markers for STEMI versus stable angina stratification that demonstrated non‐inferiority to the gold standard assay based on hs‐troponin.

Finally, the advantages of MSP as analytical probes were demonstrated in the context of EVs lacking CD9/CD63/CD81, such as RBC‐EVs, underscoring their added value to tetraspanin‐based tools and their suitability for the analysis of next‐generation bio‐nanoparticles.

In light of the aforementioned considerations, although not intended to replace the use of antibodies outright, we anticipate the widespread adoption of MSP and their integration into various isolation and analytical platforms. We speculate that, owing to its ease of use and adaptability, this technology will function as a versatile toolkit for the enrichment and analysis of extracellular vesicles (and close mimics), with the goal of seamlessly incorporating it into clinical or QC automated routines.

## 4 Experimental Section

### EV Catch and Release via Agarose Beads and Characterization

High Cobalt density agarose resins from Agarose Bead Technologies (ABT) were conjugated with MSP‐H6 peptide (6XHis‐RPPGFSPFR‐ RPPGFSPFR) as described in Benayas et al.^[^ ^31^ ^]^ For blood‐derived specimen EVs isolation, 0.1 mL of MSP‐beads suspension was added to 50 µL of sample, serum or plasma EDTA, plasma heparin, or plasma citrate diluted 1:10 in PBS, to a final volume of 500 µL, and incubated on RotoFlex for 1 h at room temperature. Using a magnetic stand, supernatant was recovered, then beads were washed three times with 0.5 mL PBS. EVs release was performed adding 100 µL of Imidazole solution 0.5 m in PBS for 15 min under shaking, at room temperature and EV suspension recovered using a magnetic stand.

### Dot and Western Blot Analysis

For analysis of contaminants, 3 µL of pure sample was dropped off on nitrocellulose membrane (Protran BA 85 Nitrocellulose, 0.45 µm, Whatman, Germany). After drying at room temperature for 15 min, the membranes were blocked with 5% of BSA in TBS containing 0.05% of Tween 20 (TBS‐T), for 1 hour. The membranes were incubated with using anti‐ApoA1 (1:1000, Santa Cruz, CA, USA), anti‐ApoE and anti‐ApoB (1:500, Santa Cruz, CA, USA), and anti‐human serum albumin (1:500, Santa Cruz, CA, USA). After washing with TBS‐T, membranes were incubated with horseradish peroxidase‐conjugated (Jackson ImmunoResearch, Tucker, GA, USA) secondary antibodies diluted 1:5000 in TBS‐T with 1% BSA for 1 h.

For Western Blot analysis of RBC‐EVs and plasma EVs, 5X Laemmli buffer was added, and samples were boiled for 5 min at 95 °C. Proteins (30 µg) were separated by SDS‐PAGE (10% polyacrylamide) and transferred onto a PVDF membrane. The blocking step was carried out with a 5% fat‐free milk in PBS‐0.05% Tween‐20 (PBS‐T) for 1 h at 37 °C. Membranes were incubated overnight at 4 °C with the following antibodies diluted in 1% fat‐free milk PBS‐T: anti‐CD9 (1:500, Santa Cruz, CA, USA), anti‐CD81 (1:500, Santa Cruz, CA, USA) anti‐CD63 (1:500, Merck‐Millipore, MA, USA), and anti‐BAND3 (1:1000, Santa Cruz, CA, USA). The membranes were washed thrice for 10 min with PBS‐T and incubated for 1 h with rabbit anti‐mouse HRP conjugated secondary antibody diluted 1:3000 in 1% fat‐free milk PBS‐T (Bethyl, TX, USA). Images were acquired with Chemibox Syngene.

### Transmission Electron Microscopy (TEM)

For negative staining 2 µL pf sample was adsorbed on a glow discharged 300 mesh formvar/carbon‐coated grids and contrasted with 2% aqueous uranyl acetate solution. Grids were air‐dried and observed with a Talos L120C (FEI, Thermo Fisher Scientific) operating at 120 kV. Images were acquired with a Ceta CCD camera (FEI, Thermo Fisher Scientific). For conventional TEM EVs absorbed on agarose magnetic beads were fixed with 2,5% glutaraldehyde in 0,1 m cacodylate buffer. Using a magnetic stand the beads were washed in cacodylate buffer and postfixed with reduced osmium (1% OsO4, 1,5% potassium ferrocyanide in 0,1 m cacodylate buffer pH 7.4) for 1 h on ice. After several washes in milli‐Q water samples were incubated in 0,5% uranyl acetate overnight at 4 °C. Samples were then dehydrated with increasing concentration of ethanol, embedded in epoxy resin, and polymerized in BEEM capsules for 48 h at 60 °C. Ultrathin sections (70–90 nm) were obtained using an ultramicrotome (UC7, Leica microsystem, Vienna, Austria), collected on copper or nickel grids, stained with uranyl acetate and Sato's lead solutions, and observed in a Transmission Electron Microscope Talos L120C (FEI, Thermo Fisher Scientific) operating at 120 kV.

### Nanoparticle Tracking Analysis (NTA)

NTA was performed according to the manufacturer's instructions using a NanoSight NS300 system (Malvern Technologies, Malvern, UK) configured with a 532 nm laser. Samples were diluted in micro‐filtered PBS; the ideal measurement concentrations were identified by pre‐testing the ideal particle per frame value (20–100 particles/frame). A syringe pump with constant flow injection was used and three videos of 60 s were captured and analyzed with Malvern NTA software version 3.4.

### SiMoA Beads Conjugation to Pan‐Tetraspanin Antibodies

Beads conjugation to antibodies was performed according to Quanterix Homebrew kit instructions using the recommended buffers as follows. Conjugation of 150 µl of carboxylate paramagnetic beads (2.8 × 10^9^ prt/ml) are washed three times with 300 µl of Bead Wash Buffer (Quanterix, phosphate buffer with detergent), after every washing step the beads are pulsed spin and placed on a magnetic separator for 1 min to aspirate the supernatant. The beads are washed three more times with 300 µl of Bead Conjugation Buffer (Quanterix, 50 mm MES buffer pH 6.2) and then are activated with EDC 0.3 mg ml^−1^ for 30 min at 4 °C under mixing/shaking.

Eighty micrograms of antibody (CD9, CD63, CD81) are buffer exchanged with a 50 KDa Amicon filter, and antibodies recovered in the Quanterix Bead Conjugation Buffer; after buffer exchange antibody concentration is measured with a Nanodrop spectrophotometer (ThermoFisher) and adjusted to 0.2 mg ml^−1^ with Bead Conjugation Buffer.

Three hundred microliter of a 0.2 mg ml^−1^ antibody solution was added to the activated paramagnetic beads and incubated for 2 h at 4 °C under mixing/shanking. After the conjugation step the beads are washed two times with Bead Wash Buffer and then are blocked with Bead Block Buffer (Quanterix, phosphate buffer with BSA) for 45 min at room temperature under mixing/shaking. After blocking, beads are washed three times with Bead Diluent and stored until used at 4 °C.

### SiMoA Beads Conjugation to MSP

One hundred and fifty microliters of SiMoA carboxylate paramagnetic beads (2.8 × 10^9^ prt/ml) were activated with EDC according to Quanterix Homebrew kit instructions as described above, then 300 µL NH_2_‐Maleimide linker (from Sigma‐Aldrich) solution 10 mm in PBS (adjusted to pH 8.6) was added and shaked for 2 h in RotoFlex. Beads were then washed two times with PBS to remove NH_2_‐ Maleimide in excess and incubated with 300 µL of 100 µm solution of MSP in PBS (adjusted to pH 8.6, with two equivalents DIEA and 1 mm TCEP). Peptide reacts for 1 h under mixing. After the conjugation step the beads were washed two times with PBS and then were blocked with Bead Block Buffer (Quanterix) for 15 min at room temperature under mixing/shaking. After blocking, beads were washed with Bead Wash Buffer (Quanterix) and stored in Bead Diluent (Quanterix) at 4 °C.

### Pan‐Tetraspanin Three‐Step Assay

Pan‐tetraspanin beads solution was prepared at the concentration of 2 × 10^7^ beads/ml in Bead Diluent. The detector antibody (biotinylated CD9, CD63, CD81 antibodies by Ancell or anti‐band 3 from Santa Cruz) solutions (0.3 µg ml^−1^) are diluted in Homebrew Sample Diluent (Quanterix); similarly, serum samples are diluted 1:4 in Homebrew Sample Diluent (Quanterix) whereas plasma samples are diluted 1:10 in Homebrew Sample Diluent. 25 µl of beads are transferred into a 96 microwell plate and mixed with 100 µl diluted sample and incubated for 30 min at 25 °C at 800 rpm. After incubation, beads were washed with an automatic plate‐washer and then incubated for 10 min with 100 µl of detector antibody After incubation, beads were washed and incubated for 10 min with a 150 pm SBG solution (in SBG Diluent, Quanterix). After SBG incubation step the plate was washed again and then inserted into the Quanterix SR‐X instrument for analysis where RGP was automatically added. Data were analyzed and processed by Reader Software Simoa 1.1.0.

### MSP SiMoA Three‐Step Assay

The assay was run as described above for pan‐tetraspanin beads except that samples and detector antibodies were incubated in PBS. The detector antibody (biotinylated CD9, CD63, CD81 antibodies or CD42a, CD62P, CD2, CD3, and CD326 by MiltenyBiotech or anti‐band 3 from Santa Cruz) was used at the concentration of 0.6 µg ml^−1^, serum samples were diluted 1:4, plasma samples were diluted 1:10. 25 µl of beads were transferred into a 96 microwell plate and mixed with 100 µl diluted sample and incubated for 30 min at 25 °C at 800 rpm. After incubation, beads were washed with an automatic plate‐washer using optimized Tween concentration and then incubated for 10 min with 100 µl of detector antibody. After that, beads were washed with an automatic plate‐washer and incubated for 10 min with a 150 pm SBG solution (in SBG Diluent, Quanterix). After SBG incubation step the plate was washed and then inserted into the Quanterix SR‐X instrument for analysis where RGP was automatically added. Data were analyzed and processed by Reader Software Simoa 1.1.0.

### Apolipoprotein Interaction Assay

The assay was run as described above for MSP beads using biotinylated anti‐ApoA1 and anti‐ApoB (Santa Cruz, CA, USA) were used at the concentration of 0.6 µg ml^−1^. Plasma was diluted 1:10. 25 µl of beads were transferred into a 96 microwell plate and mixed with 100 µl diluted sample and incubated for 30 min at 25 °C at 800 rpm. After incubation, beads were washed with an automatic plate‐washer using optimized Tween concentration and then incubated for 10 min with 100 µl of detector antibody. After that, beads were washed with an automatic plate‐washer and incubated for 10 min with a 150 pm SBG solution (in SBG Diluent, Quanterix). After SBG incubation step the plate was washed and then inserted into the Quanterix SR‐X instrument for analysis where RGP is automatically added. Data were analyzed and processed by Reader Software Simoa 1.1.0.

### Red Blood Cell Derived – EV

RBCs obtained from anonymized type 0+ healthy volunteers under written consent were provided by the blood transfusion unit of Ospedali Civili di Brescia (ethical approval NP5705) in sealed sterile bags. RBCs EVs were isolated using Ca^2+^/Ca^2+^ ionophore induction, following the guidelines from Usman et al. Briefly, RBCs were pelleted by centrifugation at 1000×g for 8 min at 4 °C, and washed thrice in sterile PBS w/o Ca^2+^ and Mg^2+^. RBCs were further washed twice with CPBS (sterile PBS + 0.1 g L^−1^ CaCl) and transferred into 175 mm^2^ tissue culture flasks. Calcium ionophore (A23187, Sigma‐Aldrich) was added to the flasks (final concentration 10 mm) and incubated overnight at 37 °C. RBCs were gently collected from the flasks, and cellular debris was removed by differential centrifugation (600×g for 20 min, 1600×g for 15 min, 3260×g for 15 min, and 10000×g for 30 min at 4 °C), discarding the pellet after each centrifugation step and transferring the supernatant into fresh sterile tubes. The supernatants were filtered through 0.45 µm nylon syringe filters (Nalgene). EVs were collected by ultracentrifugation at 50 000×g for 70 min at 4 °C. The pellets were then resuspended in cold sterile PBS, layered above a 2 mL frozen 60% sucrose cushion, and centrifuged at 50 000×g for 16 h at 4 °C, with the deceleration speed set to 0. The red layer of EVs was collected and washed twice with cold sterile PBS and spun at 50 000×g for 70 min at 4 °C. Finally, EVs were resuspended in 1 mL of cold sterile PBS, aliquoted and stored at −80 °C until used.

Centrifugations below 10 000xg were performed on an Eppendorf 5804 R equipped with a A‐4‐44 swinging bucket rotor. Ten thousand x g step was performed on a Beckman Avanti centrifuge equipped with a JA‐20 fixed angle rotor. A Beckman XPN‐80 equipped with a TY45‐Ti fixed angle rotor was employed for the ultracentrifugation step. Sucrose cushion ultracentrifugation was performed on a Beckman Optima Max‐XP equipped with a MLS‐50 swinging arms rotor. The final washing step was performed on Optima MAX‐XP equipped with a TLA‐55 rotor.

### Serum and Plasma Samples for the Clinical Validation

Peripheral venous blood samples were collected from patients recruited at the Istituto Cardiocentro Ticino, Ente Ospedaliero Cantonale (Lugano, Switzerland). The study protocol was approved by the local ethical committees. All participants gave informed written consent to the study in accordance with the declaration of Helsinki. Peripheral venous blood samples were collected from patients presenting with a diagnosis of STEMI, according to the European Society of Cardiology (ESC) guidelines^[^ ^58^ ^]^ on presentation to the emergency department before primary PCI. In addition, samples were collected from patients with chronic CAD presenting with stable angina (SA) according to ESC guidelines^[^ ^58^ ^]^ and age‐matched healthy control subjects.

For serum, blood was collected in heparin‐free polypropylene tubes, while for plasma (only in STEMI patients) in sodium citrate tubes, and centrifuged at 1600 g for 15 min at 4 °C degree to separate and discard cellular components. Serum, and free‐platelet plasma were then differentially centrifuged at 3000 g for 20 min, at 10000 g for 30 min, and at 20 000 g for 15 min as previously described;^[^ ^55^ ^]^ supernatant was aliquoted, stored at −80 °C, and never thawed prior to analysis.

### Statistical Analysis

Statistics was performed by IBM SPSS Statistics 25 (Armonk; NY) and GraphPad PRISM 9.0 (La Jolla, California). EV marker expression was compared by Kruskal–Wallis and Mann–Whitney tests. Correlations of expression levels in serum and plasma were assessed by Pearson's R test and analysis of the regression curves. The analysis of Bland‐Altman plots was used to assess the within‐sample relationship and detect systematic, proportional, or magnitude‐dependent biases. The analysis of receiver operating characteristics (ROC) curves was used to compare diagnostic performances of selected variables. A *p*‐value <0.05 was considered significant.

### Graphics

Plots were generated by Prism 9, Figures with the help of Biorender.

## Conflict of Interest

A.G. and M.C. have filed PCT/IB2020/058284 patent application titled “Conjugates composed of membrane‐targeting peptides for extracellular vesicles isolation, analysis and their integration thereof”.

## Supporting information

Supporting Information

## Data Availability

The data that support the findings of this study are available from the corresponding author upon reasonable request.
