# Supplementary material for: Addressing Heterogeneity in Direct Analysis of Extracellular Vesicles and Their Analogs by Membrane Sensing Peptides as Pan‐Vesicular Affinity Probes
Source: Adv Sci (Weinh). 2024 May 31;11(29):2400533. doi: 10.1002/advs.202400533 (PMC11304302; doi:10.1002/advs.202400533)
Supplement: Supplementary file 1 — Supporting Information [file ADVS-11-2400533-s001.docx]

**Addressing Heterogeneity in Direct Analysis of Extracellular Vesicles and their Analogues by Membrane Sensing Peptides as Pan-vesicular Affinity Probes**

Alessandro Gori^1*§^, Roberto Frigerio^1§^, Paola Gagni^1^, Jacopo Burrello^2^, Stefano Panella^2^, Andrea Raimondi^3^, Greta Bergamaschi^1^, Giulia Lodigiani^1^, Miriam Romano^4,5^, Andrea Zendrini^4,5^, Annalisa Radeghieri^4,5^, Lucio Barile^2,6^ and Marina Cretich^1*^

*1: Consiglio Nazionale delle Ricerche, Istituto di Scienze e Tecnologie Chimiche “Giulio Natta” (SCITEC), Milano, Italy*

*2: Cardiovascular Theranostics, Istituto Cardiocentro Ticino, Ente Ospedaliero Cantonale, Via Tesserete 48, CH-6500, Bellinzona, Switzerland*

*3: Institute for Research in Biomedicine, Faculty of Biomedical Sciences, Università della Svizzera italiana (USI), CH-6500, Bellinzona, Switzerland*

*4: Department of Molecular and Translational Medicine, University of Brescia, Viale Europa 11, 25123 Brescia, Italy*

*5: CSGI, Center for Colloid and Surface Science, 50019 Florence, Italy*

*6: Euler Institute, Faculty of Biomedical Sciences, Università della Svizzera Italiana, 6900 Lugano, Switzerland*

§: Equally contributed

*: Corresponding authors

marina.cretich@cnr.it

alessandro.gori@cnr.it

**Supplementary Information**

**Figure S1:** Immune dot-blot for the evaluation of Alipopoprotein A, Apolipoprotein B, Apolipoprotein E and serum albumin in starting sample, supernatant, release fraction. Blood was collected from healthy subjects, and four preanalytical conditions evaluated: serum, Plasma EDTA, Plasma Citrate, Plasma heparin. Plasma and serum were isolated in parallel from the same subject. EDTA, heparin and citrate tubes were used for the collection of plasma, while serum was obtained in clot activator tubes. Two centrifuge steps were performed for all samples: 1500g for 10 minutes and 2500g for 10 minutes

**Table S1**

Raw AEB data and %CV for experiments reported in Figure 3.

**MSP conjugation onto SiMoA beads**

MSP conjugation to beads via cysteine-maleimide click reaction was monitored by RP-HPLC. A calibration curve of MSP-Cys was used for quantification of peptide in the conjugation mixture (starting solution 100uM peptide concentration) and in the remaining supernatant after incubation with maleimide activated beads. The area under the curve (AUC) was measured for each run and estimated MSP concentrations were extrapolated. The reproducibility of the conjugation was monitored in four conjugation batches leading to average conjugation efficiency of 96.43% ± 2%.

**Figure S2. A):** Scheme of immunocapturing experiments in plasma samples. SiMoA paramagnetic beads were functionalized with MSP, as well as with individual CD63, CD9, and CD81 antibodies. Beads were incubated with a pool of 6 plasma samples according to SiMoA three step assay reported in Materials and Methods except that the supernatant was analyzed after each incubation by immune-dot blot analysis. Two cycles of immunocapturing were performed. **B):** Dot blot analysis for the EV markers Mitofillin, CD41, MHC II and TSG101 in the plasma reference and in supernatant after the first and second cycle of immunocapturing.


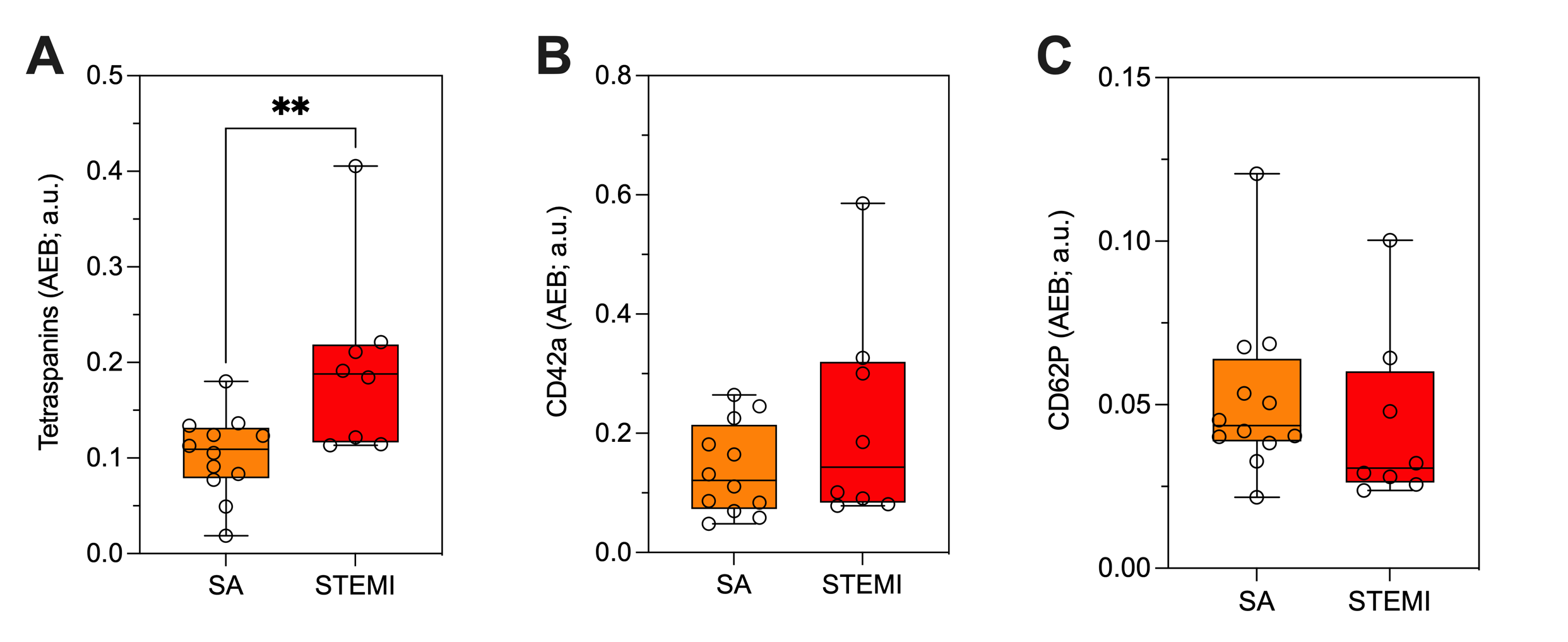


**Figure S3: A)** Expression of Tetraspanins CD9/CD81/CD63, **B)** CD42a and **C**) CD62P in plasma of patients with ST-segment elevation myocardial infarction (STEMI; red, n=12), stable angina (SA; orange, n=12). t test : Tetraspanins - *p*= 0.009 CD42a - *p*= 0.343 CD62P - *p*= 0.238

The study was conducted with MSP modified beads in a customized SiMoA assay as described in the Materials and Methods Section.


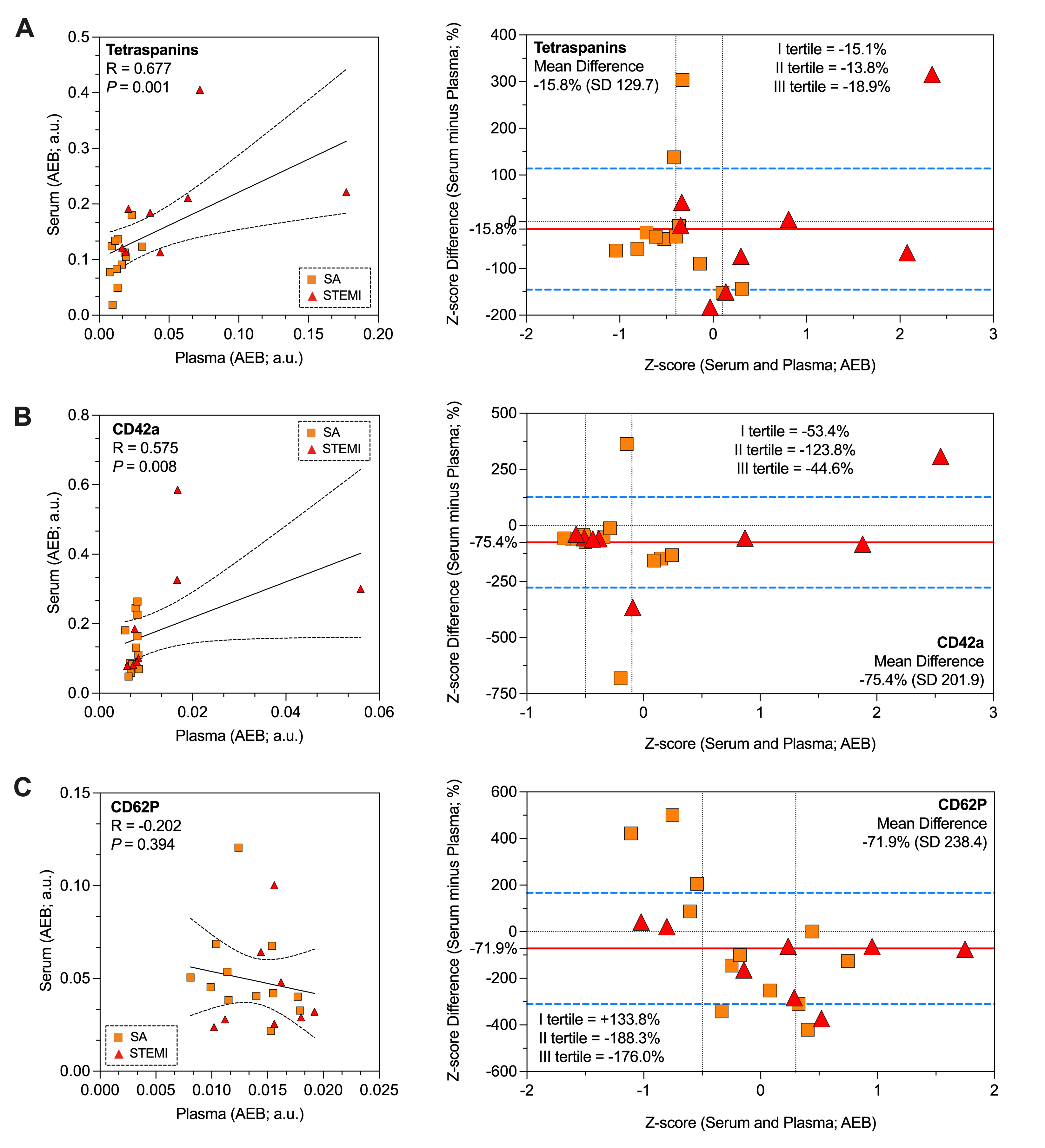


**Figure S4**: Expression levels in serum and plasma were correlated by Pearson’s R test in patients with STEMI or SA (n=12; left column). Bland Altman plots evaluating serum and plasma expression of tetraspanins (**A**) CD42a (**B**) and CD62P (**C**) after normalization by Z-score (n=12; right column). Difference between serum and plasma expression levels is reported on Y-axis; mean expression in serum and plasma is reported on X-axis, for each EV marker. The red line indicates mean percentage underestimation of expression levels in serum compared to matched plasma samples, together with 95% confidence interval (blue dotted lines); tertiles of expression in serum and plasma are marked on the X-axis, together with the mean difference of serum minus plasma in each of them
